# Effect of the chives, pomegranate, and lime juices on the meat quality and microbial reduction of chicken breast inoculated with *Salmonella* Enteritidis

**DOI:** 10.1007/s44463-025-00019-8

**Published:** 2026-04-16

**Authors:** Joko Sujiwo, Hee-Jin Kim, Hye-Jin Kim, Aera Jang

**Affiliations:** 1 Department of Applied Animal Science, Kangwon National University, Chuncheon, 24341 Korea; 2 Poultry Research Center, National Institute of Animal Science, Rural Development Administration, Pyeongchang, 25342 Korea; 3 Center for Food and Bioconvergence, Seoul National University, Seoul, 08826 Korea; 4 Department of Animal Production, Faculty of Animal Science, Universitas Gadjah Mada, 55281 Sleman, Yogyakarta, Indonesia

**Keywords:** Meat preservation, *Salmonella* Enteritidis, Plant juice, Antimicrobial, Antioxidant

## Abstract

The antimicrobial and antioxidant properties of lyophilized juice from chives (*Allium tuberosum* R., CVJ), pomegranate (*Punica granatum*, PGJ), and lime (*Citrus aurantifolia*, LMJ) were evaluated in this study. These lyophilized juices were used as natural preservatives for chicken breast artificially inoculated with *Salmonella enterica* serovar Enteritidis over 9 days at 4 °C. LMJ demonstrated the least minimum inhibitory concentration at 50 mg/mL, while the minimum bactericidal concentration for CVJ and LMJ was 100 mg/mL, and for PGJ, it was 200 mg/mL. In the 1,1-diphenyl-2-picrylhydrazyl assay, PGJ exhibited the strongest antioxidant capacity with the lowest IC_50_ value (2.17 mg/mL). According to the ferric-reducing antioxidant power assay, LMJ exhibited the greatest antioxidant capacity, with an EC_50_ value of 0.68 mg/mL. Meat inoculated with *S.* Enteritidis and then treated with 1% CVJ (SCVJ), 1% PGJ (SPGJ), or 1% LMJ (SLMJ) had lower pH values than the control from the fifth day of storage onwards (*p* < 0.05). On day 5, SCVJ had significantly lower *S.* Enteritidis counts (5.16 Log CFU/g) compared to the control (5.46 Log CFU/g). SCVJ had significantly lower volatile basic nitrogen levels than the other treatments by day 7, followed by SPGJ and SLMJ with values of 25.09, 26.84, and 28.28 mg/100 g, respectively. SLMJ effectively maintained lower 2-Thiobarbituric acid reactive substances values. Among the treatments, SLMJ yielded the most favorable results, preserving meat quality without significant color changes.

## Introduction

Global demand for poultry meat, especially chicken, is projected to increase significantly, 15.9 kg/cap/year consumption of poultry in 2019 is projected to rise in 2050 by 26.2 kg/cap/year globally (Falcon et al., 2022). Chicken is high in protein, low in fat and contains relatively high omega-3 polyunsaturated fatty acids. Widely accepted by religions and valued for its neutral taste, a versatile and cost-effective protein. However, chicken meat tends to have a short shelf life and deteriorate over time. This can be primarily attributed to the presence of high levels of unsaturated fat and spoilage microorganisms from its natural microbiota and the slaughterhouse environment. When stored at 4 °C, freshly slaughtered chicken meat lasts about 4–6 days (Musalem et al., 2024).

Chicken meat is prone to bacterial contamination during production, transport, and storage, leading to rapid spoilage and foodborne illness. Foodborne illness can be attributed to pathogens including strains of *Listeria monocytogenes*, *Escherichia coli*, *Salmonella* spp., and *Campylobacter* spp. (Kang et al., 2022). *Salmonella* spp. represents a significant proportion of the most prevalent foodborne pathogens observed in poultry products. Poultry industries struggle to inactivate *Salmonella* spp. while preserving meat quality, making pathogen control a major challenge (Dirks et al., 2012).

Common methods, such as heat treatments, bacteriocins, lactic acid bacteria, and chemical agents, can reduce microbes but may also harm meat’s nutritional and sensory qualities (Kim et al., 2002). Nonthermal methods like ultrasonication, irradiation, high-pressure processing, and plasma effectively control microbes without heat-induced damage, however they have limitations such as shallow penetration and uneven effects (Lee et al., 2023). Consumer demand for clean-label, naturally preserved foods has increased markedly, driving growth in the global natural preservatives market. This trend has intensified the search for natural antimicrobial agents suitable for poultry processing (Grant & Parveen, 2017). Natural preservatives offer a promising means of ensuring food safety while addressing concerns about synthetic additives. Utilizing compounds derived from herbs and fruits presents an affordable, sustainable strategy that aligns with market preferences and may provide additional nutritional benefits. An emerging trend in the consumer market has been the growing demand for natural food preservatives, driven by increasing consumer awareness of health and sustainability concerns (Novais et al., 2022). The use of herbs and fruits as natural material offers numerous advantages, including ease of implementation, affordability, and nutritional benefits.

The genus *Allium*, which includes chives (*Allium tuberosum* Rottler), has a long history in ethnomedicine and culinary practice. Their antibacterial properties make them effective food preservatives. A research shows that *Allium* species can inhibit bacterial growth in meat products, which increases their preservation time and improves quality (Sharifi-Rad et al., 2016). Pomegranate (*Punica granatum*) is another natural compound shown to effectively preserve meat, with its antimicrobial activity attributed to organic acids, polyphenols, punicalagin, and punicalin (Rasuli et al., 2021). Howell and D’Souza (2013) reported that pomegranate demonstrated antimicrobial activity against foodborne pathogens, including *Salmonella*. Similar to chives and pomegranate, lime (*Citrus aurantifolia*) juice, rich in acids, essential oils, saponins, and flavonoids, exhibits antimicrobial properties which extend the meat shelf life of culled layer hens (Suradi et al., 2015). Despite these promising attributes, there remains a paucity of studies evaluating lyophilized (freeze-dried) chive, pomegranate, and lime juices as natural preservatives specifically against *Salmonella enterica* serovar Enteritidis in chicken meat.

Therefore, this study aims to characterize the antimicrobial and antioxidant activities of lyophilized chive, pomegranate, and lime juices and assess their efficacy in extending the shelf life of chicken breast experimentally inoculated with *S.* Enteritidis under refrigerated storage.

## Materials and methods

### Plant juice preparation

Samples of chives (*Allium tuberosum* R.), pomegranates (*Punica granatum*), and limes (*Citrus aurantifolia*) were procured locally on a commercial market in Chuncheon, Korea. Before juicing, the samples underwent a thorough washing process with clean and running water. The pomegranate and lime peels were removed before the juicing. A single batch of plant juice was prepared by juicing the fresh material, followed by lyophilization. Fresh ingredients were pressed using a juicer (NJE-3834; NUC Electronics, Daegu, Korea), yielding both liquid and solid residues. Only the liquid portion, free of insoluble dregs, was collected, and the remaining solids discarded. The collected juice was then lyophilized and stored at −20 °C until use in subsequent experiments. The lyophilization yield was 4.71% w/w for chive juice (CVJ), 11.53% w/w for pomegranate juice (PGJ), and 9.50% w/w for lime juice (LMJ). It is important to note that no biological replicates were included in the preparation of the juice. Consequently, the results obtained reflect the inherent technical variation present within a single batch. Although only a single juice batch was used, all assays were performed in triplicate (technical replicates, *n* = 3) to ensure analytical reliability.

### Antimicrobial activity of plant juice

#### Preparation of *Salmonella* Enteritidis inoculum

The *Salmonella enterica* serovar Enteritidis (CCARM 8260) was retrieved from the Culture Collection of Antibiotic Resistant Microbes, Seoul, South Korea. Stock cultures were revived by streaking onto Brilliant Green Sulfa (BG Sulfa) agar and Xylose Lysine Deoxycholate (XLD) agar plates (MBCell, Seoul, Korea), which were incubated at 37 °C for 24 h. A single presumptive colony from the selective plates was then subcultured onto Tryptic Soy Agar (TSA) (MBCell, Seoul, Korea) and incubated under the same conditions to ensure purity. One colony from the TSA plate was aseptically transferred into 10 mL of Tryptic Soy Broth (TSB) (MBCell, Seoul, Korea) and incubated at 37 °C for 24 h. Thereafter, 100 µL of this overnight culture was inoculated into fresh 10 mL TSB and incubated at 37 °C for 18 h. The resulting culture was used for both antimicrobial activity assays and artificial contamination of chicken breast samples.

### Paper disc diffusion analysis

The lyophilized plant juice was solubilized in distilled water (DW) at concentrations of 1.25, 2.5, 5, or 10 milligrams per disc. Preparation of the inocula entailed the transfer of a single loopful of bacterial culture to 10 mL of TSB, followed by incubation for 24 h at 37 °C. Following this, the culture was grown to a target concentration of 5 to 6 Log CFU/mL, then it was seeded onto TSA. The 8-mm paper discs (Toyo Roshi Kaisha, Ltd., Tokyo, Japan) utilized in this study were prepared in a sterile manner. These discs were then placed on the TSA surface, and 50 µL of samples were dispensed onto each disc. The negative control comprised 50 µL DW applied to a paper disc, while 0.2 mg/disc streptomycin constituted the positive control. The plates were then subjected to an incubation process at 37 °C for 24 h. Subsequently, the inhibition zone diameters were measured. This process was replicated in triplicate.

### Minimum inhibitory concentration (MIC) and minimum bactericidal concentration (MBC) analysis

To determine the MIC and MBC, the TSB was utilized in 96-well plates through the implementation of the microdilution method. The assay involved the addition of 130 µL of TSB, along with 20 µL of a microbial mixture and an additional 50 µL of the sample, to individual wells of a microtiter plate. The range of sample concentrations that was tested was from 0 to 200 mg/mL, with a 200 µL final volume in individual wells. Subsequent to this, the prepared plates underwent an incubation at 37 °C for 24 h. Thereafter, the degree of turbidity was measured at a wavelength of 600 nm, utilizing a spectrophotometer (SpectraMax M2e, Molecular Devices, USA). To determine the MIC, the lowest sample concentration that inhibited visible growth in the broth was recorded. The MBC values were determined by removing the bacterial suspension from subculture, which demonstrated no visible growth, and then inoculating it onto TSA plates. Subsequently, an incubation at 37 °C for 24 h was initiated. The MBC is defined as the least sample concentration capable of eradicating 99.9% of the bacterial inocula.

### Antioxidant activity of the plant juice

#### Measurement of 1, 1-diphenyl-2-picrylhydrazyl (DPPH) radical scavenging activity

A 100 µL solution of a 0.2 mM DPPH mixture was combined with 100 µL of the sample solution, subsequently placed in a 96-well microplate, and incubated in the dark at room temperature for a duration of 30 min. The measurement was conducted using a spectrophotometer at a wavelength of 517 nm. Trolox was utilized as a positive control. The percentage of DPPH scavenging activity was calculated using this formula:

$$\text{DPPH radical scavenging activity (\%)} =1 - \frac{\text{sample O.D.} - \text{reference O.D.}}{\text{control O.D.}} \times 100$$

Control = 100 µL 0.2 DPPH mM solution in MeOH + 100 µL DW.

Reference = 100 µL MeOH + 100 µL DW.

The concentration that provided 50% inhibition (IC_50_) was determined by calculating the y-intercept of the line plotted “DPPH radical scavenging activity” (x-axis) against concentration (y-axis). In essence, lower IC_50_ values are indicative of higher antioxidant activity.

#### The 2,2′-azinobis-3-ethylbenzothiazoline-6-sulfonic acid (ABTS) scavenging activity measurement

A method developed by Re et al. (1999) was employed to assess ABTS scavenging activity. A mixture of 7 mM ABTS and 2.45 mM potassium persulfate was prepared, and it underwent a 12–16 h dark storage period at ambient temperature. Subsequently, the mixture was diluted to achieve an optical density of 0.700 (± 0.002) at 735 nm. A volume of 950 µL of the ABTS solution was combined with 50 µL of the sample, and the mixture was subsequently incubated at 30 °C for 30 min. Spectrophotometric analysis was then conducted to measure the sample’s absorption at a wavelength of 735 nanometers. The radical scavenging activities were expressed as IC_50_ values. The IC_50_ value corresponds to the concentration of the sample required for 50% inhibitory effect on ABTS radical scavenging activity.

### Ferric reducing antioxidant power (FRAP) evaluation

Utilizing the method outlined by Benzie and Strain (1996), the FRAP assessment ensued. The preparation of the FRAP reagent involved the mixture of acetate buffer (300 mM, pH 3.6), 2,4,6-tripyridyl-S-triazine (TPTZ) (10 mM in 40 mM HCl), FeCl_3_·6H_2_O (20 mM) in a 10:1:1 (v/v) ratio. An aliquot of 25 µL of the sample was added to 175 µL of the FRAP reagent, and the mixture was incubated for 30 min at 37 °C. The optical density was measured at a wavelength of 590 nm using a spectrophotometer. Absorbance values indicated the extent of reducing power in the sample. The effective concentration (EC_50_ in mg/mL) was determined from the linear regression analysis.

### Oxygen radical absorbance capacity (ORAC) evaluation

To perform the ORAC assay, slight modifications were made to the method outlined by Gillespie et al. (2007). A total of 25 µL of sample and 150 µL of fluorescein (80 nM) were vortexed followed by incubation at for 15 min 37 °C. Subsequently, AAPH (2,2’-azobis(2-methylpropionamidine) dihydrochloride) (150 mM) was added, resulting in a total volume of 200 µL. Fluorescence measurements were then obtained using a spectrophotometer at 480 nm and 520 nm wavelengths, with readings collected at one-minute periods for a duration of 60 min. This was followed by the plate being subjected to automatic shaking at 37 °C. The resulting data were presented as the EC_50_, which was determined through the implementation of linear regression analysis.

### Treatment of plant juices on chicken breasts artificially inoculated with *Salmonella* Enteritidis

Fresh, skinless chicken breasts were procured from a local slaughterhouse in Chuncheon, Korea immediately after slaughter. The samples were transported to the laboratory on ice. Under sterile conditions, the samples were inoculated with *S.* Enteritidis (6–7 Log CFU/mL). The inoculation process involved dipping the samples in TSB and sterile saline solutions for 15 min. After inoculation, samples were placed in a sterile, clean bench for 20 min at room temperature to allow colonization and drying. A total of 60 chicken breast samples were randomly assigned to four treatment groups (*n* = 15 per group). For each group, three independent samples (biological replicates, *n* = 3), sourced from different chicken carcasses, were collected and analyzed at designated storage days (days 0, 3, 5, 7, and 9) to reflect typical chilled poultry product shelf-life. The chicken breast meat inoculated with *S.* Enteritidis were treated by dipping the sample for 15 min in an aqueous solution of either 1% CVJ (SCVJ), 1% PGJ (SPGJ), 1% LMJ (SLMJ), or DW as a control (SCON). The 1% concentration for the juice solutions was chosen over 3% and 5% based on preliminary trials and practical considerations for meat preservation. Although higher concentrations exhibited stronger antimicrobial activity, they also caused undesirable changes in appearance. Specifically, levels above 1% produced greenish hues in CVJ and excessive redness in PGJ, as well as surface sliminess and lingering odors, all of which could undermine consumer acceptance. Additionally, this concentration was chosen in alignment with the methodology of Saleh et al. (2022), who utilized 1% of lime juice concentration in their study. Following the dipping treatment, a drying period at 25 ± 2 °C for 20 min was implemented under aseptic conditions. The samples subjected to treatment were then arrayed on styrofoam plates, covered with low-density polyethylene plastic wrap, and preserved at 4 °C. All measurements for each sample-including microbial counts, pH, water holding capacity (WHC), color (CIE L*a*b*), volatile basic nitrogen (VBN), 2-thiobarbituric acid reactive substances (TBARS), and shear force-were conducted in triplicate (technical replicates) to ensure analytical accuracy and reproducibility.

### Measurement of chicken breast pH value

An Orion 230 A pH meter (Thermo Fisher Sci. Inc., Waltham, MA, USA) was utilized to measure the pH values. A homogenate was prepared by blending 90 mL of DW with 10 g of the sample for 30 s using a homogenizer (Polytron R PT-2500 E, Kinematica, Switzerland). After blending, the pH measurement was conducted by inserting the pH meter probe into the homogenate.

### Measurement of chicken breast water holding capacity (WHC)

A 0.5 g portion of the sample was heated in a water bath at 80 °C for 20 min, followed by cooling at room temperature for 10 min. The sample was then centrifuged at 2,000×g for 20 min at 4 °C to assess water loss. The percentage of water loss in relation to the total moisture content of the sample was calculated to determine the WHC.

$${\text{WHC (\%) = }}\frac{{{\text{total moisture - water loss}}}}{{{\text{total moisture}}}}{{ \times 100}}$$

$$\begin{aligned}\text{Water loss} &=\frac{\text{weight before centrifugation} - \text{weight after centrifugation}}{\text{sample weight} \times \text{fat factor}} \times 100\end{aligned}$$

$${\text{Fat factor = 1}} - \frac{{{\text{crude fat (\%)}}}}{{{\mathrm{100}}}}$$

### Measurement of chicken breast instrumental color

Colorimetric analysis was employed to assess the instrumental meat color, utilizing a CR-300 colorimeter (Minolta Co., Osaka, Japan). The CIE 1976 (L*a*b*) color space was employed to quantify the color values of lightness, redness, and yellowness. These values were measured at multiple locations on the surface of the meat.

### Total aerobic bacteria (TAB) and *Salmonella* Enteritidis cell count of chicken breast determination

Each 10 g chicken meat sample was aseptically placed into individual stomacher bags containing 90 mL of sterile saline solution and homogenized in a stomacher (Bag Mixer 400 P, Interscience, France) for 40 s. Serial dilutions of each sample prepared in sterile saline solution were then obtained. Subsequently, appropriate dilutions of the sample mixtures were applied onto dry media. After an incubation at 37 °C for 48 h, the total aerobic bacteria were enumerated using plate count agar (PCA; Difco Laboratories, Detroit, USA). The enumeration of *S.* Enteritidis was performed by spreading the homogenates onto selective xylose lysine deoxycholate (XLD; MB Cell Ltd., Seoul, Korea) agar plates. The populations of *S.* Enteritidis were determined after the plates were incubated for 48 h at 35 °C.

### Chicken breast volatile basic nitrogen (VBN) content measurement

Precisely 10 g of grounded sample and 50 mL of DW were thoroughly homogenized for 30 min with a magnetic stirrer. This mixture was filtered through filter paper (Whatman No. 1). A 1-mL sample filtrate was added to the outer chamber of the Conway unit, while an additional 1-mL solution of 0.01 N H_2_SO_4_ was introduced into the inner chamber. Finally, 1 mL of K_2_CO_3_ was loaded into the opposing outer chamber. The chamber was then promptly covered, clipped, and sealed. The sample underwent an incubation at 25 °C for 1 h. Post-incubation, a solution of Brunswik indicator was added to the inner chamber and the titration was conducted with 0.01 N NaOH. An equation was used to calculate the VBN content:

Volatile Basic Nitrogen (mg/100 g) = 0.14 × (b - a) × F/W × 100 × d.

Where:

- a = Volume of the sample added in 0.01 N NaOH (mL).
- b = Volume of the blank added in 0.01 N NaOH (mL).
- F = Standard factor of 0.01 N NaOH.
- W = Sample weight (g).
- d = Dilution factor.

### Chicken breast 2-Thiobarbituric acid reactive substances (TBARS) assay

Five grams of chicken meat samples were added to 50 µL of 7.2% BHT and 15 mL of DW, which were subsequently mixed for 30 s using a homogenizer. Then, 1 mL of the homogenized meat sample was placed into a test tube, followed by the addition of 2 mL of thiobarbituric acid/trichloroacetic acid (TBA/TCA). Subsequently, 20 mM TBA and 15% TCA were administered. One milliliter of DW and two milliliters of the TBA/TCA solution were used as the blank. The sample was then vortexed and subjected to a 15 min incubation process at 90 °C in a boiling water bath. Following this, ice-water was used to sample cooling, then subjected to a centrifugation at 4 °C, 2000×g for 10 min. The resulting supernatant was analyzed for its absorbances at 531 nm. To express the TBARS values, the results were presented as milligrams of malondialdehyde (MDA) per kg of meat. A standard equation was used to calculate the TBARS value:

TBARS (mg MDA/kg sample) = (Absorbance of sample - Absorbance of blank) × 5.88.

### Measurement of chicken breast shear force

The sample was submitted to a constant temperature water bath, set to 75 °C ± 2 °C for 45 min, prior to the application of shear force. Subsequent to the heating process, the meat samples were sliced into 1 × 3 × 2 cm pieces. Using a Texture Analyzer TA 1 (Lloyd Instruments, Berwyn, PA, USA), shear force values were measured. Configuration settings for this instrument included a load cell of 50 kg, 50 mm/min test speed, 50 mm/min trigger speed, and 10 gf trigger force.

### Statistical analysis

All antimicrobial and antioxidant assay results for plant juices are expressed as the mean and standard error of the mean (SEM) from three technical replicates (*n* = 3), whereas meat quality parameters are based on biological replicates. Data were analyzed using one-way analysis of variance (ANOVA) with a general linear model in SAS software version 9.2 (SAS Institute, Cary, NC, USA).  Significant differences among treatment means were determined using Tukey’s multiple comparison test, withstatistical significance at *p* < 0.05.

## Results and discussion

### Antimicrobial and antioxidant activity of plant juices

**Fig. 1:**
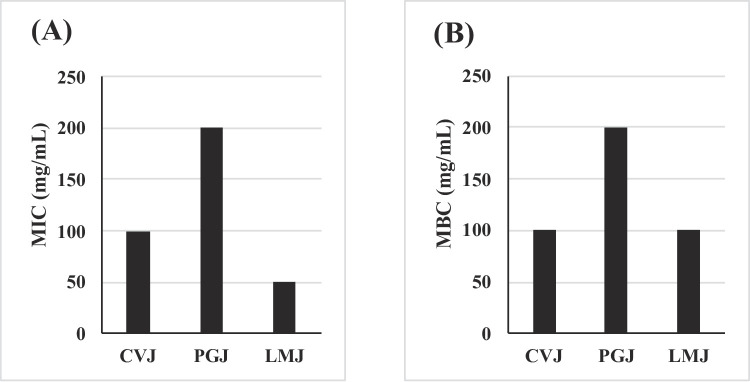
Minimum inhibitory concentrations (MIC, A) and minimum bactericidal concentrations (MBC, B) of plant juices against *Salmonella* Enteritidis CVJ, chives juice; PGJ, pomegranate juice; LMJ, lime juice

The antimicrobial activity of a lyophilized juice against *S.* Enteritidis was evaluated using paper disc diffusion, MIC, and MBC methods. The results of the paper disc assay were presented as the zone of inhibition (clear zone) in mm diameter (Table 1). CVJ showed no inhibition at 2.5 or 5 mg/disc but produced a moderate inhibition zone of 10.44 mm at 10 mg/disc, slightly exceeding the positive control (9.67 mm). Similarly, PGJ showed no activity at lower concentrations but exhibited an inhibition zone of 10.11 mm at 10 mg/disc, comparable to the positive control (10.00 mm). In contrast, LMJ demonstrated superior antimicrobial activity, with zones of inhibition exhibited 11.11 mm at 5 mg/disc and 12.67 mm at 10 mg/disc, significantly larger than the positive control (9.33 mm) (*p* < 0.05). The antibacterial activity of CVJ, PGJ, and LMJ in this study were classified as sufficient or moderate based on inhibition zone diameters. Antibacterial activity was categorized as follows: ≥ 20 mm (very strong), 15–19 mm (strong), 10–14 mm (moderate), and < 10 mm (weak) (Kim et al., 2020b). The inhibition zone of lime juice observed in this study is comparable to Fadillah et al. (2020) findings who found 7–15 mm against *Salmonella* Typhi. For MIC (Fig. 1A), CVJ required 100 mg/mL, PGJ showed the highest MIC at 200 mg/mL, and LMJ demonstrated the lowest MIC at 50 mg/mL, indicating superior inhibitory efficacy. The MBC values (Fig. 1B) followed a similar trend: CVJ and LMJ both required 100 mg/mL, whereas PGJ exhibited the highest MBC of 200 mg/mL, suggesting lower bactericidal effectiveness compared to the other juices. Lime juice demonstrated the strongest antimicrobial performance, achieving the largest inhibition zone, lowest MIC and relatively low MBC. Lime juice exhibited antibacterial activity through membrane disruption, acidification, and the introduction of bioactive compounds that suppress bacterial growth and metabolism (Suradi et al., 2015).

**Table 1:** Antimicrobial activity of plant juices against *Salmonella* Enteritidis using disc diffusion assay (mm)

Plant juices	2.5 mg/disc	5 mg/disc	10 mg/disc	PC	SEM
CVJ	ND	ND	10.44	9.67	0.314
PGJ	ND	ND	10.11	10.00	0.343
LMJ	ND	11.11^b^	12.67^a^	9.33^c^	0.231

^a−c^ Means within the same row with different letters are significantly different (*p* < 0.05)

CVJ, chives juice; PGJ, pomegranate juice; LMJ, lime juice. PC, Positive control (streptomycin, 0.2 mg/disc). ND, the zone of inhibition was not detected

Table 2 presents the lyophilized plant juices antioxidant activity measured by various assays, including DPPH, ABTS, FRAP, and ORAC, represented by IC_50_ and EC_50_ values. For the DPPH assay, PGJ exhibited the strongest antioxidant activity with the lowest IC_50_ value (2.17 mg/mL), followed by LMJ (4.91 mg/mL) and CVJ (7.96 mg/mL). To clearly demonstrate the antioxidant potential of the tested juices, DPPH radical scavenging activity was further analyzed at a fixed concentration of 1 mg/mL, enabling a direct comparison among treatments. At this concentration, PGJ showed the highest scavenging activity (34.85%), followed by LMJ (26.09%) and CVJ (23.15%). This ranking is in line with the IC_50_ values reported in Table 2, where a lower IC_50_ indicates stronger antioxidant capacity. These results highlight PGJ as having the most potent DPPH radical scavenging ability among the three types of plant juice.

**Table 2:** Antioxidant activity of plant juices

Plant juices	IC_50_ (mg/mL)		EC_50_ (mg/mL)	
	DPPH	ABTS	FRAP	ORAC
CVJ	7.96^A^	7.81^A^	2.62^A^	0.10^C^
PGJ	2.17^C^	4.08^C^	2.65^A^	0.30^A^
LMJ	4.91^B^	6.81^B^	0.68^B^	0.16^B^
SEM	0.349	0.261	0.035	0.010

^A−C^ Means within the same column with different letters are significantly different (*p* < 0.05)

CVJ, chives juice; PGJ, pomegranate juice; LMJ, lime juice. SEM, standard errors of the mean. IC_50_, the concentration of juice required to scavenge 50% of the initial free radicals. EC_50_, the concentration of juice required to achieve 50% of the maximum antioxidant effect

Similar trends were observed in the ABTS assay, where PGJ again showed superior activity (4.08 mg/mL) compared to LMJ (6.81 mg/mL) and CVJ (7.81 mg/mL). The FRAP assay revealed that LMJ had the highest reducing power (0.68 mg/mL), significantly outperforming CVJ (2.62 mg/mL) and PGJ (2.65 mg/mL). Lastly, the ORAC assay demonstrated that PGJ had the highest antioxidant capacity (0.30 mg/mL), followed by LMJ (0.16 mg/mL) and CVJ (0.10 mg/mL). The results indicate that PGJ demonstrates the most robust overall antioxidant activity, particularly in radical-scavenging assays like DPPH and ABTS, likely due to its high polyphenol content, particularly punicalagin (Gil et al., 2000). Lime juice demonstrated exceptional reducing power in the FRAP assay, suggesting its capability to reduce of ferric ions to ferrous ions (Azman et al., 2019). However, CVJ exhibited the weakest antioxidant activity across all assays, despite the presence of organosulfur compounds. Based on antimicrobial and antioxidant evaluations, the juices demonstrate potential for treating *S.* Enteritidis-inoculated chicken breast.

A limitation of the study is that variability across batches such as differences in cultivation period, or regional origin was not assessed, as only a single batch of each juice was analyzed. To mitigate analytical variance within this batch, all assays were performed using three technical repeats (technical replication), which enhanced confidence in the precision and reproducibility of the measured outcomes by identifying and reducing random experimental error. Nevertheless, future studies incorporating multiple independent juice batches will be required to evaluate the consistency of both yield and bioactivity across variable conditions.

### pH value and water holding capacity of chicken breast

Table 3 presents the pH values of inoculated chicken breast which are treated with various lyophilized plant juices. The pH value during storage of the chicken breast was gradually increased whether on the control group or the treatment group. An initial observation revealed that the pH value of the chicken breast exhibited significant variation among the treatment and control groups. Although the difference was moderate, according to the statistical analysis it was found to be statistically significant. This minimal difference may be related to the use of a single batch of plant juice in the study. The lower pH value observed in SPGJ and SLMJ samples was likely due to the presence of organic acids in these juices. The pH value of pomegranate juice ranged around 3.00 to 3.30 (Miguel et al., 2004) and 2.16 to 2.77 for lime juice (Ogundele & Bolade, 2021). Pomegranates contained citric and malic acids, while limes are rich in citric acid (Penniston et al., 2008). The initial phase of the storage period was designated as day 0, SLMJ had a significantly lower pH (*p* < 0.05) compared to the other treatments, followed by SPGJ, SCON, and SCVJ, which showed pH values of 5.97, 6.01, 6.07, and 6.10, respectively. A significantly lower pH value of the treatment group was observed on days 5, 7, and 9 of storage compared to the control group. The control group showed a consistent increase in pH, reaching the highest value (6.68) by day 9. Among the plant juice treatments, SPGJ maintained the lowest pH throughout storage, starting at 6.01 and ending at 6.31 on day 9. Lime juice (SLMJ) also effectively controlled pH, exhibiting values consistently lower than the control but slightly higher than SPGJ by the end of the storage process. The higher pH value indicated that the deterioration of the meat was initiated when the pH value surpassed 6.20 (Kim et al., 2019). The increase in pH can be attributed to the production of alkaline compounds, such as ammonia, by Enterobacteriaceae along with other related microorganism, which leads to muscle alkalinization during the storage process (Orkusz et al., 2024). The phenomenon is attributable to the degradation of amino acids followed by the formation of ammonia through proteolysis (Marareni et al., 2024).

**Table 3:** Effect of plant juice solution on pH value and water holding capacity of chicken breast meat inoculated with *Salmonella* Enteritidis during storage at 4 °C

Treatment	Storage days					SEM
	0	3	5	7	9	
pH value						
SCON	6.07^Bd^	6.19^Acd^	6.28^Abc^	6.44^Ab^	6.68^Aa^	0.036
SCVJ	6.10^Ad^	6.16^ABc^	6.20^Bc^	6.31^BCb^	6.39^Ba^	0.010
SPGJ	6.01^Cb^	6.07^ABb^	6.10^Cb^	6.23^Ca^	6.31^Ba^	0.021
SLMJ	5.97^Dd^	6.01^Bc^	6.04^Dc^	6.32^Bb^	6.41^Ba^	0.007
SEM	0.005	0.037	0.007	0.018	0.025	
Water holding capacity (%)						
SCON	77.28	75.62	76.37	72.88	71.95	3.622
SCVJ	75.61	73.33	76.81	76.08	74.09	5.033
SPGJ	74.02	75.50	77.95	75.40	74.58	4.952
SLMJ	74.93	74.82	76.40	73.11	70.61	5.194
SEM	2.808	5.025	5.149	4.249	5.894	

^A−D^ Means within the same column with different letters are significantly different (*p* < 0.05)

^a−d^ Means within the same row with different letters are significantly different (*p* < 0.05)

SCON, the inoculated breast meat with *S.* Enteritidis without any plant juice treatment (control); SCVJ, the breast meat inoculated with *S.* Enteritidis treated with 1% CVJ (chives juice); SPGJ, the breast meat inoculated with *S.* Enteritidis treated with 1% PGJ (pomegranate juice); SLMJ, the breast meat inoculated with *S.* Enteritidis treated with 1% LMJ (lime juice)

Lowering pH during meat storage creates a preservation environment that both inhibits microbial growth and affects physicochemical properties, influencing safety and quality. Acidic conditions disrupt microbial function by collapsing the proton motive force, which is vital for energy production and nutrient uptake. This leads to an energy crisis that hampers bacterial growth. Low pH also denatures proteins, inactivating key enzymes such as ATPase and DNA polymerase (Gaucher et al., 2019). Additionally, H⁺ ions alter membrane integrity by interacting with phospholipids, increasing permeability and causing leakage of cellular contents (Sun et al., 2024).

The WHC value of chicken breast did not significantly change for the treatment and control groups (Table 3). In addition, the WHC of all samples exhibited no significant alterations during the extended period of storage. The WHC levels for chicken breast in this study ranged from 73.11% to 77.95%. In line with Wang et al. (2022) study, which found that chicken breast’s WHC ranged from 69.47% to 82.06%.

### Instrumental meat color of chicken breast

Table 4 presents the CIE L*, a*, and b* color values of the *S.* Enteritidis-inoculated chicken breast that was then dipped in the various plant juice solutions and refrigerated at 4 °C. The CIE L* value of all samples gradually decreased with the prolonged storage time. On day three of storage, the CIE L* value of the SCON declined dramatically, while the SCVJ and SPGJ declined on day five of storage. However, SLMJ could maintain the CIE L* value until day seven of storage. Up to day 5 of storage, there was no discernible difference between the treatment and control groups in terms of the CIE L* value. The extended color stability conferred by LMJ compared to the other treatments likely reflects its antioxidant activity, which helps preserve the visual appeal of refrigerated chicken meat. Frazão et al. (2024) similarly reported that lime’s major components, D-limonene and β-pinene, are responsible for this color-preserving effect in meat. Consistent with their findings, our study demonstrated that LMJ also effectively inhibits lipid oxidation, a key driver of meat discoloration during storage.

**Table 4:** Effect of plant juice solution on the color of chicken breast meat inoculated with *Salmonella* Enteritidis during storage at 4 °C

Trait	Treatment	Storage days					SEM
		0	3	5	7	9	
L*	SCON	62.70^Aa^	59.47^Ab^	59.82^ABab^	56.85^Cb^	56.78^ABb^	0.736
	SCVJ	62.52^Aa^	61.28^Aa^	58.12^Bb^	58.57^Bb^	55.58^ABc^	0.460
	SPGJ	60.83^Aa^	60.38^Aab^	58.26^Bb^	53.39^Dc^	54.16^Bc^	0.583
	SLMJ	61.45^Aa^	60.76^Aa^	61.46^Aa^	60.67^Aa^	58.00^Ab^	0.551
	SEM	0.577	0.668	0.582	0.429	0.666	
a*	SCON	1.71^Ba^	1.69^Ba^	1.64^Ba^	1.57^Ba^	1.68^Ba^	0.086
	SCVJ	−3.04^Ca^	−3.19^Ca^	−3.03^Ca^	−3.04^Ca^	−3.05^Ca^	0.162
	SPGJ	2.57^Aa^	2.50^Aa^	2.57^Aa^	2.31^Aa^	2.43^Aa^	0.137
	SLMJ	1.87^Ba^	1.83^Ba^	1.85^Ba^	1.87^ABa^	1.89^Ba^	0.113
	SEM	0.119	0.157	0.147	0.131	0.114	
b*	SCON	3.53^Bd^	5.40^Bc^	5.90^Bbc^	6.60^Bab^	7.14^Ba^	0.221
	SCVJ	8.76^Ab^	9.36^Ab^	10.81^Aa^	11.51^Aa^	11.52^Aa^	0.231
	SPGJ	3.48^Be^	4.27^Cd^	5.36^Bc^	6.09^Bb^	6.93^Ba^	0.168
	SLMJ	3.29^Bd^	4.49^Cc^	5.70^Bb^	5.85^Bb^	7.41^Ba^	0.187
	SEM	0.119	0.205	0.221	0.221	0.228	

^A−D^ Means within the same column with different letters are significantly different (*p* < 0.05)

^a−e^ Means within the same row with different letters are significantly different (*p* < 0.05)

SCON, the inoculated breast meat with *S.* Enteritidis without any plant juice treatment (control); SCVJ, the breast meat inoculated with *S.* Enteritidis treated with 1% CVJ (chives juice); SPGJ, the breast meat inoculated with *S.* Enteritidis treated with 1% PGJ (pomegranate juice); SLMJ, the breast meat inoculated with *S.* Enteritidis treated with 1% LMJ (lime juice)

The CIE a* value of the chicken breast on all samples did not significantly change during storage for 9 days. The SCVJ exhibited the lowest CIE a* value in comparison to the other treatments over the course of the storage period. The low CIE a* value of the SCVJ was due to the greenish color of the chicken breast. The greenish color of SCVJ came from the green color of chives. On the other hand, over the period of storage, SPGJ’s CIE a* value was noticeably higher than that of the other treatments. Pomegranate juice’s red hue might have been the cause of the high SPGJ CIE a* score.

The CIE b* value of the chicken breast, irrespective of whether it was part of the control or the treatment groups, gradually increased with prolonged storage time. Similarly, Sujiwo et al. (2018) reported that the b* value of chicken breast increased by the fifth day of storage. The CIE b* value for SCVJ was significantly higher compared to other treatments. This elevated CIE b* value in SCVJ might be attributed to the yellow-greenish hue imparted by the natural color of chives. There was no significant difference among SCON, SPGJ, and SLMJ across storage days 0, 5, 7, and 9.

**Fig. 2:**
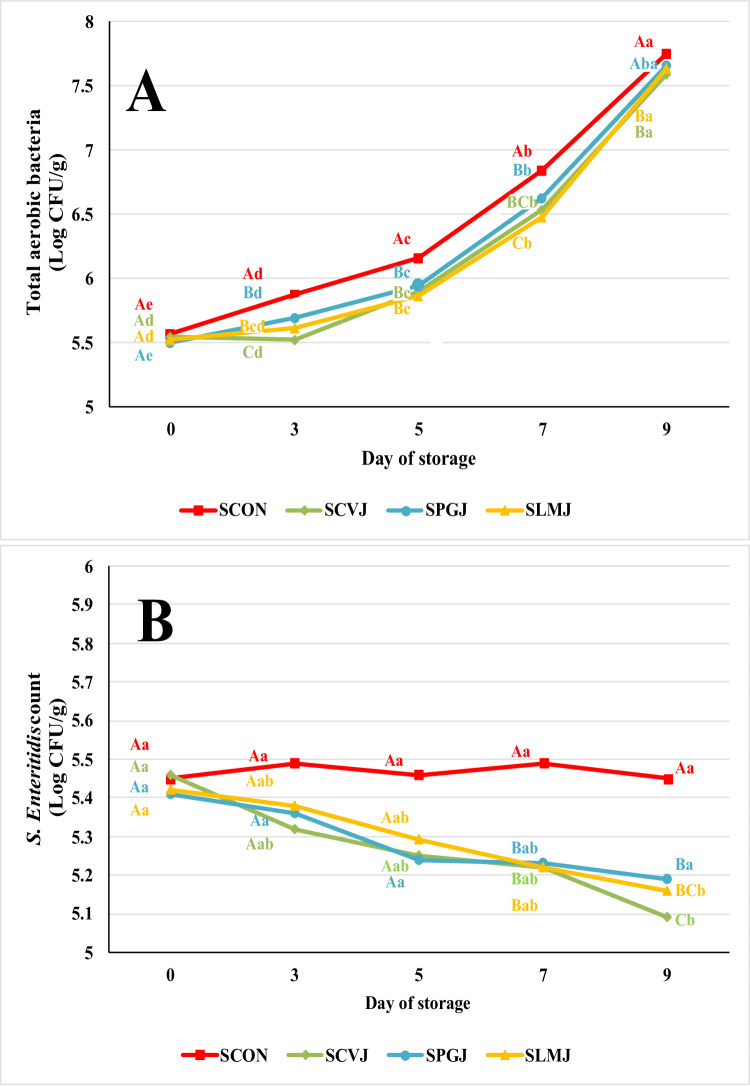
Effect of plant juice on the total aerobic bacteria (**A**) and *Salmonella* Enteritidis (**B**) of chicken breast meat inoculated with *Salmonella* Enteritidis during storage at 4 °C SCON, the inoculated breast meat with *S.* Enteritidis without any plant juice treatment (control); SCVJ, the breast meat inoculated with *S.* Enteritidis treated with 1% CVJ (chives juice); SPGJ, the breast meat inoculated with *S.* Enteritidis treated with 1% PGJ (pomegranate juice); SLMJ, the breast meat inoculated with *S.* Enteritidis treated with 1% LMJ (lime juice). ^A−C^ Values of the bar with different superscript among treatments differ significantly at *p* < 0.05. ^a−e^ Values of the bar with different superscripts among days differ significantly at *p* < 0.05

The CIE L* and CIE a* values of the SCON and SLMJ in this study were comparable to those observed by Sujiwo et al. (2018), which reported a CIE L* value range of 58.30 to 59.06 and an CIE a* value range of 0.91 to 1.00. In addition, during storage, they found that the CIE b* value of the chicken breast was around 3.66 to 5.73. In the current study, the CIE b* value of SCON and SLMJ ranged from 3.29 to 7.41, which is marginally higher than the values reported by Sujiwo et al. (2018). The discoloration of meat during storage is attributable to oxidized myoglobin, which converts myoglobin to metmyoglobin, giving the meat a brownish color (Kim et al., 2019). The transformation of myoglobin (Mb) to metmyoglobin (metMb) occurs through the oxidation of the iron atom in the heme group from its ferrous (Fe²⁺) to ferric (Fe³⁺) state (Arcon et al., 2015). Nonetheless, due to its lower myoglobin content, poultry meat does not undergo significant discoloration like beef (Min et al., 2010). Microbial growth during storage also contributes to meat discoloration by producing metabolites that can accelerate the oxidation of myoglobin to metmyoglobin (Wang et al., 2021). This study showed that during the storage the population of microorganisms was significantly increased (Fig. 2A). Consistent with (Ribeiro et al., 2019), our findings suggest natural antioxidant applications effectively preserve color stability particularly the SLMJ.

While natural antioxidants like chives and pomegranate juices effectively curb lipid oxidation in meat, their high-intensity pigments can induce undesirable color shifts potentially conflicting with consumer expectations. Similar to previous findings, incorporating the extract of green vegetables negatively altered the color of the meat products (Kim et al., 2013). To address this, strategies such as optimizing concentrations, encapsulating pigments to delay release, or blending with mild synthetic antioxidants could balance preservation and visual appeal. Consumer education on the link between natural preservatives and freshness may also recalibrate preferences. Future work should prioritize sensory testing to define acceptable color thresholds.

### Total aerobic count (TAB) and *Salmonella* Enteritidis cell count of chicken breast

The initial TAB counts of the meat on day 0 of storage ranged from 5.50 to 5.57 Log CFU/g, showing no significant difference among the samples (Fig. 2A). Over the storage period of 9 days, the TAB counts in all samples gradually increased. However, the SCVJ and SLMJ exhibited significantly lower TAB counts compared to the control group on days 3, 5, 7, and 9 of storage. The antimicrobial effects of chives and lime juices helped in suppressing the growth of aerobic bacteria, effectively slowing down the spoilage process. However, it should be noted that TAB counts in treated samples reached approximately 5.52–5.58 Log CFU/g by day 3, approaching the acceptable limit of 6.70 Log CFU/g set by the Korean Ministry of Food and Drug Safety (MFDS, 2018). Thus, although SCVJ and SLMJ delayed bacterial growth relative to control, storage beyond 3 days may not be acceptable from a safety or quality standpoint without additional preservation measures or comprehensive sensory evaluation.

On days 0 and 3 of storage, there was no discernible difference in the initial population of *S.* Enteritidis on chicken breast meat between the treatment and control groups (Fig. 2B). However, on day 5, *S.* Enteritidis counts of the SCVJ group (5.16 Log CFU/g) were noticeably lower than those of the control group (5.46 Log CFU/g) and other treatments. With no discernible variations across the treatment groups, the *S.* Enteritidis counts in the chicken breast dipped in plant-juice media (SCVJ, SPGJ, and SLMJ) decreased significantly compared to the control group on days 7 and 9 of storage. Over the course of the storage period, the *S.* Enteritidis population in the control group stayed mostly constant, whereas it progressively declined in the treatment groups.

Even though the effect was subtle, a statistically significant (*p* < 0.05) microbial reduction was observed compared to the control. The highest reduction in TAB counts was 0.36 log CFU/g, observed for both SCVJ (day 3) and SLMJ (day 7). For *S.* Enteritidis, the highest reduction was observed with SCVJ on day 9 (0.36 log CFU/g), followed by SLMJ, which showed a reduction of 0.29 log CFU/g. This subtle effect may be attributed to one batch preparation and low concentration (1%) of plant juice used in this study. This concentration was chosen based on initial experiments, which showed that concentrations above 1% led to severe alterations in the appearance of chicken breast. These appearance changes may be attributable to the use of crude juice. Therefore, it is proposed that purified or concentrated extracts be used in future studies so that higher levels of bioactive compounds can be achieved and antimicrobial efficacy improved without adversely affecting appearance.

Chive juice exhibits antimicrobial activity due to allicin-derived organosulfur compounds such as ajoene, DAS, and DADS (Bhatwalkar et al., 2021). Allicin induces thiol stress by forming S-allylmercapto adducts with cysteine residues in bacterial proteins. This modification irreversibly alters protein conformation, as demonstrated in pathogenic bacteria where allicin treatment depleted glutathione levels by 97% and modified critical enzymes like isocitrate lyase AceA (Müller et al., 2016). Similarly, the high content of quinones, tannins, terpenoids, phenolic acids, and polyphenols in pomegranate and lime juices played a significant role in suppressing microbial growth. The phenolic compounds in these juices may inhibit microbial growth through mechanisms such as enzyme inhibition or protein binding, contributing to the reduced *S.* Enteritidis counts in SPGJ and SLMJ samples (Vaithiyanathan et al., 2011).

The use of plant juice solutions, particularly chives, pomegranate, and lime juices, demonstrated effective antimicrobial properties, making them practical choices for improving the safety and prolonging the shelf life of chicken meat. However, we concede that only a single *Salmonella enterica* serovar Enteritidis strain was used in this study, and that stress tolerance and antimicrobial susceptibility can vary among different strains and serovars. Therefore, our findings are specific to the strain tested and may not fully represent the behavior of other *Salmonella* strains. Future studies should evaluate a multi-strain inoculum, including both clinically relevant and food-derived serovars, to assess the generalizability of plant juice–mediated inhibition across diverse *Salmonella* populations.

### Volatile basic nitrogen (VBN) and 2-Thiobarbituric acid reactive substances (TBARS) values of chicken breast

Up until the third day of storage, there was no discernible change in the VBN values of the treatment and control groups (Table 5). Nonetheless, the VBN values of the treated samples were significantly (*p* < 0.05) lower than those in the control group on days 5 to 9. On day 7, the VBN value of SCVJ showed markedly lower values compared to the other treatments, followed by SPGJ and SLMJ, with values of 25.09, 26.84, and 28.28 mg/100 g, respectively. VBN values in all samples gradually increased as storage time progressed. Sujiwo et al. (2018) reported a strong correlation between VBN values and microbial population growth, which aligns with the gradual increase in microbial populations observed during storage in this study. VBN provides an index of muscle food freshness, reflecting protein breakdown products resulting from microbial and enzymatic activity. During the storage of meat, the presence of microorganisms and endogenous enzymes can lead to the degradation of protein molecules, leading to the accumulation of nitrogenous compounds such as ammonia, trimethylamine, and dimethylamine, collectively measured as VBN (Bekhit et al., 2021). The recommended limit for VBN levels, in terms of meat palatability, is below 20 mg/100 g (Kim et al., 2019).

**Table 5:** Effect of plant juice solution on VBN and TBARS value of chicken breast meat inoculated with *Salmonella* Enteritidis during storage at 4 °C

Treatment	Storage days					SEM
	0	3	5	7	9	
VBN (mg/100 g)						
SCON	11.18^Ad^	11.39^Ad^	14.52^Ac^	31.97^Ab^	43.68^Aa^	0.307
SCVJ	10.85^Ac^	11.50^Ac^	12.35^Bc^	25.09^Cb^	35.28^Ca^	0.307
SPGJ	11.20^Ac^	11.46^Ac^	12.70^Bc^	26.84^BCb^	39.02^Ba^	0.463
SLMJ	10.71^Ac^	11.27^Ac^	12.23^Bc^	28.28^Bb^	40.88^Ba^	0.580
SEM	0.402	0.475	0.258	0.578	0.467	
TBARS (mg MDA/kg)						
SCON	0.11^Ad^	0.14^Ac^	0.16^Ac^	0.18^Ab^	0.21^Aa^	0.004
SCVJ	0.11^Ad^	0.12^Bcd^	0.14^Bbc^	0.15^Bb^	0.18^Ba^	0.005
SPGJ	0.11^Ad^	0.13^ABcd^	0.13^Bbc^	0.15^Bb^	0.17^Ba^	0.003
SLMJ	0.11^Ac^	0.12^Bbc^	0.13^Bbc^	0.14^Bb^	0.18^Ba^	0.004
SEM	0.005	0.003	0.004	0.004	0.004	

^A−C^ Means within the same column with different letters are significantly different (*p* < 0.05)

^a−d^ Means within the same row with different letters are significantly different (*p* < 0.05)

SCON, the inoculated breast meat with *S.* Enteritidis without any plant juice treatment (control); SCVJ, the breast meat inoculated with *S.* Enteritidis treated with 1% CVJ (chives juice); SPGJ, the breast meat inoculated with *S.* Enteritidis treated with 1% PGJ (pomegranate juice); SLMJ, the breast meat inoculated with *S.* Enteritidis treated with 1% LMJ (lime juice)

The TBARS values, which serve as an indicator of lipid oxidation of chicken breast meat inoculated with *S.* Enteritidis, are shown in Table 5. Across all samples, TBARS values gradually increased with prolonged storage. In the control group (SCON), a significant increase in TBARS was observed on day 3 of storage, whereas in SCVJ and SPGJ, the increase was delayed until day 5, and in SLMJ, until day 7. No differences in the initial TBARS values were found between the control and treatment groups. Nevertheless, from days three to nine, TBARS levels of SCVJ and SLMJ were noticeably lower than SCON. The TBARS values of SCVJ on days 3, 5, 7, and 9 showed 0.12, 0.14, 0.15, and 0.18 mg MDA/kg, respectively, while those of SLMJ were 0.12, 0.13, 0.14, and 0.18 mg MDA/kg, respectively. On the other hand, TBARS levels of SCON on the same days were 0.14, 0.16, 0.18, and 0.21 mg MDA/kg. As shown in Table 2, the significant antioxidant activity of lime and chives is responsible for the reduced TBARS levels in SCVJ and SLMJ-treated samples. Chives are known for their sulfur-driven radical scavenging properties, which stem from the presence of sulfur compounds such as allyl sulfides. These compounds neutralize free radicals by donating hydrogen atoms, effectively interrupting the chain reactions involved in lipid peroxidation. Moreover, they chelate pro-oxidant metals like iron and copper, preventing the occurrence of Fenton reactions that produce highly reactive hydroxyl radicals (Sharifi-Rad et al., 2020). The polyphenols present in pomegranates, including anthocyanins, ellagitannins, and punicalagins, have been shown to regulate oxidation processes by stabilizing heme proteins, donating electrons to radicals, and inhibiting pro-oxidative enzymes (Mahdavi & Javadivala, 2021). Meanwhile, the citric acid found in lime juice has been observed to chelate metal ions, while ascorbic acid plays a role in regenerating antioxidants (Novais et al., 2022). These processes utilize pH reduction to suppress oxidative enzyme activity. The production of lipid hydroperoxides, which break down into secondary products including ketones, aldehydes, and alcohols, is linked to elevated TBARS levels and causes unpleasant flavors and odors in meat (Shahidi & Hossain, 2022). Several studies have reported acceptable TBARS thresholds for consumers, ranging from 0.4 to 1.0 mg MDA/kg (Choe et al., 2019; Kim et al., 2020a). Regardless of *S.* Enteritidis contamination, the chicken breast meat in this investigation satisfied acceptability standards based only on TBARS values. Furthermore, the application of dipping treatments with plant juices led to a substantial decrease in the rate of lipid oxidation, underscoring the efficacy of these juices as natural antioxidants in maintaining the quality of meat products.

### The chicken breast’s shear force value

After nine days of storage at 4 °C, no discernible variations in the shear force values between the treatment and control groups were found (Table 6). However, a decreasing trend in shear force values was noted with prolonged storage. On day 0, the shear force values ranged from 28.94 to 30.31 N, while on day 9, they ranged from 13.73 to 16.78 N. These findings align with a previous study, which reported shear force values for chicken breast meat ranging from 18.04 to 31.38 N (Oliveira et al., 2015). The findings showed that longer storage times result in more tender meat, as shown by lower shear force values. A reliable method for assessing the tenderness of meat involves measuring shear force. This tenderness increase is likely due to the degradation of myofibrillar proteins, which can result from enzymatic activity or microbial processes. The observed trends may be linked to the rise in TAB counts during storage (Fig. 2A), which could contribute to a protein breakdown and a subsequent decrease in shear force values.

**Table 6:** Effect of plant juice solution on shear force (N) value of chicken breast meat inoculated with *Salmonella* Enteritidis during storage at 4 °C

Treatment	Storage days					SEM
	0	3	5	7	9	
SCON	30.12^a^	25.31^ab^	22.17^bc^	19.23^cd^	15.50^d^	0.107
SCVJ	29.72^a^	24.62^ab^	23.84^bc^	19.23^cd^	16.78^d^	0.114
SPGJ	30.31^a^	27.47^ab^	22.86^bc^	19.42^cd^	13.73^d^	0.133
SLMJ	28.94^a^	25.21^ab^	22.66^bc^	19.52^cd^	14.91^d^	0.103
SEM	0.154	0.139	0.051	0.117	0.083	

^a−d^ Means within the same row with different letters are significantly different (*p* < 0.05)

SCON, the inoculated breast meat with *S.* Enteritidis without any plant juice treatment (control); SCVJ, the breast meat inoculated with *S.* Enteritidis treated with 1% CVJ (chives juice); SPGJ, the breast meat inoculated with *S.* Enteritidis treated with 1% PGJ (pomegranate juice); SLMJ, the breast meat inoculated with *S.* Enteritidis treated with 1% LMJ (lime juice)

## Conclusion

This study demonstrated that immersing chicken breast in solutions of plant juices, particularly SLMJ, contributes to preserving meat quality and modestly reduces the contamination of *S.* Enteritidis. Although SCVJ and SLMJ exhibited statistically significant reductions compared to the control (*p* < 0.05), the observed inhibition of TAB and *S.* Enteritidis growth was minimal. Despite this limited antimicrobial impact, SLMJ offered notable benefits in other areas. It preserved the quality of the meat without causing significant color changes and controlled the pH value more effectively than control. By the end of the storage period, the *S.* Enteritidis population in SLMJ remained significantly lower than in the control group, though the difference was minimal. While SCVJ was more effective in maintaining a lower VBN value, SLMJ also exhibited a significantly lower VBN value compared to the control. Additionally, SLMJ maintained a lower TBARS value, suggesting reduced lipid oxidation. These findings indicate that, although the antimicrobial effect of lime juice is modest, its broader contributions to meat quality preservation make it a potentially valuable organic agent for enhancing the safety and shelf life of chicken meat. Furthermore, because only a single *Salmonella enterica* serovar Enteritidis strain was evaluated, the broader application to other *Salmonella* serovars and strains are needed. Another limitation of this study is the lack of comprehensive sensory evaluation of the treated meat, which could not be conducted due to the inoculation with *S.* Enteritidis. Moreover, the use of a single batch of plant juices may have led to subtle differences among the samples. Future research should involve multiple independent preparations of plant juices and their application without pathogen contamination to enable sensory evaluation and assess consumer perception. For commercial adoption, optimizing plant juice concentrations by advance extraction and application methods can balance antimicrobial efficacy with sensory impact. While pilot-scale trials should standardize protocols to ensure uniform coverage without modification on meat appearance. Additionally, sourcing extracts from low-cost agricultural by-products and comparing costs with synthetic preservatives could enhance economic viability, and scalable techniques like freeze-drying or encapsulation may stabilize bioactive for industrial use.
